# Covid-19 lockdown: Ethnic differences in children’s self-reported physical activity and the importance of leaving the home environment; a longitudinal and cross-sectional study from the Born in Bradford birth cohort study

**DOI:** 10.1186/s12966-021-01183-y

**Published:** 2021-09-06

**Authors:** Daniel D. Bingham, Andy Daly-Smith, Jennifer Hall, Amanda Seims, Sufyan A. Dogra, Stuart J. Fairclough, Mildred Ajebon, Brian Kelly, Bo Hou, Katy A. Shire, Kirsty L. Crossley, Mark Mon-Williams, John Wright, Kate Pickett, Rosemary McEachan, Josie Dickerson, Sally E. Barber, the Bradford Institute for Health Research COVID-19 Scientific Advisory Group

**Affiliations:** 1 Bradford Institute for Health Research, Bradford Teaching Hospitals NHS Foundation Trust, Bradford Royal Infirmary, Duckworth Lane, Bradford, BD9 6RJ UK; 2 Faculties of Life Sciences and Health Studies, University of Bradford, Richmond Road, Bradford, BD7 1DP UK; 3 Centre for Applied Education Research, Wolfson Centre for Applied Health Research, Bradford Royal Infirmary, Bradford, West Yorkshire UK; 4 Health Research Institute and Movement Behaviours, Health, and Wellbeing Research Group, Department Sport and Physical Activity, Edge Hill University, Ormskirk, L39 4QP UK; 5 Department of Health Sciences, SeebohmRowntree Building, University of York, University of York, Heslington, YO10 5DD York UK; 6 School of Psychology, University of Leeds, Leeds, West Yorkshire UK

**Keywords:** COVID-19, Lockdown, Physical activity, Children, Ethnicity, Moderate-to-vigorous, Self-report, Correlates, Environment

## Abstract

**Background:**

In England, the onset of COVID-19 and a rapidly increasing infection rate resulted in a lockdown (March-June 2020) which placed strict restrictions on movement of the public, including children. Using data collected from children living in a multi-ethnic city with high levels of deprivation, this study aimed to: (1) report children’s self-reported physical activity (PA) during the first COVID-19 UK lockdown and identify associated factors; (2) examine changes of children’s self-reported PA prior to and during the first UK lockdown.

**Methods:**

This study is part of the Born in Bradford (BiB) COVID-19 Research Study. PA (amended Youth Activity Profile), sleep, sedentary behaviours, daily frequency/time/destination/activity when leaving the home, were self-reported by 949 children (9–13 years). A sub-sample (n = 634) also self-reported PA (Physical Activity Questionnaire for Children) pre-pandemic (2017-February 2020). Univariate analysis assessed differences in PA between sex and ethnicity groups; multivariable logistic regression identified factors associated with children’s PA. Differences in children's levels of being sufficiently active prior to and during the lockdown were examined using the McNemar test; and multivariable logistic regression was used to identify factors explaining change.

**Results:**

During the pandemic, White British (WB) children were more sufficiently active (34.1%) compared to Pakistani Heritage children (PH) (22.8%) or ‘Other’ ethnicity children (O) (22.8%). WB children reported leaving the home more frequently and for longer periods than PH and O children. Modifiable variables related to being sufficiently active were frequency, duration, type of activity, and destination away from the home environment. There was a large reduction in children being sufficiently active during the first COVID-19 lockdown (28.9%) compared to pre-pandemic (69.4%).

**Conclusions:**

Promoting safe extended periods of PA everyday outdoors is important for all children, in particular for children from ethnic minority groups. Children’s PA during the first COVID-19 UK lockdown has drastically reduced from before. Policy and decision makers, and practitioners should consider the findings in order to begin to understand the impact and consequences that COVID-19 has had upon children’s PA which is a key and vital behaviour for health and development.

**Supplementary Information:**

The online version contains supplementary material available at 10.1186/s12966-021-01183-y.

## Background

In England, the immediate response to the first wave of Severe Acute Respiratory Syndrome Coronavirus 2 (SARS-CoV-2)—COVID19—pandemic was a stringent lockdown implemented on 23^rd^ March 2020 [1]. The government placed extreme restrictions on movement of the public stating that *“during the emergency period, no person may leave the place where they are living without reasonable excuse”,* which included shopping for food and medical supplies [1]*. *Furthermore, guidance stipulated that members of the public could also leave the home for a short bout (60 min) of local daily exercise. All playgrounds and indoor and outdoor play facilities (e.g. skate parks, soft play centres) were closed, in addition to leisure facilities and gyms. Schools were closed for most children with the exception of vulnerable children and children of key workers (those working across health, social and public sectors). The lockdown measures were eased in England on 4^th^ July 2020 [2]. However, at the time of writing, two further national lockdowns have occurred in England, in November 2020 [3] and January 2021 [3, 4].

Before the COVID-19 pandemic, national and international epidemiological data (whether device or self-reported measured) report that up to 80% of children and young people in high-income countries are not sufficiently physically active for health and well-being (e.g. achieve 60 min of MVPA per day) [5, 6]. Within England, recent survey data suggests that the 53.2% of children, aged 5–16 years, were not achieving physical activity (PA) guidelines [7]. Of specific concern, levels of inactivity were higher in children from ethnic minority groups, especially those with South Asian heritage [8, 9]. Such low levels of PA place children at risk of poor physical and mental wellbeing in addition to having a negative impact on school performance [10–14]. Within South Asian communities, such risks are high as children present with increased rates of Obesity and type-II diabetes [15]. Further, early evidence suggest such populations are more likely to suffer the most during and after the pandemic [4, 16–20]. Research conducted during COVID-19 has already reported low levels and significant reductions of children's PA [21, 22]. It is essential to understand the impact of the pandemic on PA levels and behaviours for different ethnic groups for two reasons, first to prevent inactive behaviours becoming entrenched and second, to tailor support for different populations by addressing the root causes of PA inequality within different populations [23].

The Born in Bradford (BiB) research programme [24] provides a premium opportunity to study the impact of COVID-19 lockdown on school-aged children living in a deprived and ethnically diverse city. To date, BiB has tracked/monitored the health, wellbeing, and determinants of health of over 30,000 Bradford residents (parents and children) since 2007 [24]. The latest round of data collection occurred pre COVID (2017-March 2020, n = 7500, aged between 6–11 years) [25], establishing a pre-COVID baseline; providing a unique opportunity to understand the impact of the COVID lockdown on physical activity behaviour in an ethnically diverse sample of school-aged children and young people. Further, the BiB cohort study will follow participants throughout the duration of the pandemic and in the following years, to understand the impact of the crisis on health and wellbeing trajectories [26].

The current study is part of the wider Born in Bradford COVID-19 Research Study [26] and aims to: 1) report children’s self-reported physical activity (PA) during the first COVID-19 UK lockdown and identify associated factors; 2) examine changes of children’s self-reported PA prior to and during the first UK lockdown.

## Methods

### Setting

Bradford is the fifth largest metropolitan district in England with a population of 530,000 [24]. It is an ethnically diverse city situated in the North of England, with almost half of the births in the city are to women of South Asian (mostly Pakistani) heritage [27–29]. Levels of poverty and ill health (including cardiovascular disease and diabetes) in Bradford are some of the highest in England, and a large proportion of households are classed as overcrowded [28]. Almost a quarter of Bradford children live in poverty and 25% are living with obesity at age 11, and the rates of childhood obesity are 6% higher among the same age group of South Asian children compared to White British children [28]. Such socio-economic and structural characteristics of Bradford make the community particularly vulnerable to COVID-19.

### Participants and procedure

Participants were children aged 9–13 who were invited to take part in the BiB COVID-19 research study [26] following a protocol approved by the Health Research Authority and Bradford/Leeds research ethics committee (reference: 16/YH/0320).

The parents/carers of 5,298 children aged 9–13 years who are participants in the existing BiB birth cohort study and who had engaged in a recent follow-up data collection wave pre-COVID-19 (2017-early March 2020) [25], were contacted by trained researchers via telephone to invite their child to take part in a survey. Following verbal consent from parents/carers, children received a survey via post to be completed and returned to the research team using pre-paid envelopes [26]. Completion of the survey was deemed as participation assent from the child. Overall, 970 children returned surveys during the period of May 21st to July 31st 2020 and 949 children (97.8%) provided enough data to be included for the analysis of the first two aims of this research.

### Measures

#### Demographic measures

Children’s age, sex, ethnicity, and home postcode-derived Index of Multiple Deprivation (IMD) [29] were extracted from the BiB cohort dataset. Three categories of ethnicity were used for the analysis, White British (WB), Pakistani Heritage (PH) (the two largest groups in the sample) and ‘Other’ (O) (any other ethnic group). Index of Multiple Deprivation (IMD) deciles [30] were categorised into either the ‘most deprived nationally’ (most deprived 10% areas in England), ‘2^nd^ most deprived nationally’ (10–20% most deprived areas), ‘3^rd^ most deprived nationally’ (30–40% most deprived areas), and ‘4^th^ or more most deprived nationally’ (40%-100% most deprived areas). Child’s school attendance during the April-June 2020 lockdown period was included in the survey.

#### During COVID-19 Lockdown

During the first COVID-19 lockdown PA, sedentary behaviours, screen-time, sleep, activity (frequency, duration, type, and place) away from home environment were all measured by child self-report (Table 1). Self-reported PA was measured using a modified version of the validated seven day recall questionnaire, the Youth Activity Profile- English Youth Version (YAP) [31, 32]. The YAP requires children to report the frequency and/or duration of physical activities through different segments of a usual day (i.e. before school, break time at school, lunch at school, after school). During the first lockdown most children were not attending school, so this format was not appropriate, and for the same reason, neither was the PA questionnaire for children (PAQ-C) [33] which was the questionnaire completed by children in data collection pre-COVID [25]. The choice to amend the YAP, which was originally based upon the PAQ-C, and to not use the PAQ-C was due to the YAP specifically including an item asking directly for an estimation of time in overall MVPA across weekend days. Following consultation with the lead author of the English child version of the YAP (also a co-author of this study-SF) a decision was made to ask the YAP-weekend item along with an overall weekday item, using the same wording (Table 1). The YAP was also used to estimate sedentary behaviours whilst watching television, playing video games, using a mobile phone, a computer/tablet during COVID-19 restrictions. An additional question of ‘*doing school work*’ was also included to capture the amount of time children spent doing sedentary school work during COVID-19 restrictions (Table 1). A binary variable of meeting screen time (ST) guidelines (< 2 h a day) [34, 35] was calculated by the values of each answer for sedentary screen behaviours (Table 1). Children’s average sleep time was estimated by children reporting the time they normally go to sleep and the time they normally wake up. Sleep time was categorised into meeting sleep guidelines [34] (9-to-11 h a day) or more or less.

**Table 1:** Questionnaire items and processing methods used for during COVID-19 first lockdown analysis

Variable(s)	Question(s)	Response option	Processing variable	Source
Sufficiently physically active (normally doing 60 min of moderate-vigorous physical activity everyday)	a) for a normal weekday (Monday, Tuesday, Wednesday, Thursday, Friday), during the last 7 days, how much physical activity did you do? (e.g. dancing, online exercise, games/sports, jobs at home, cycling). This can be anything that made you feel warmer, breathe harder or your heart beat faster.’ b) for a normal weekend day (Saturday, Sunday), during the last 7 days, how much physical activity did you do? (e.g. dancing, online exercise, games/sports, jobs at home, cycling). This can be anything that made you feel warmer, breathe harder or your heart beat faster.’	a) No activity (0 min) b) small amount activity (1 to 30 min) c) small to moderate amount of activity (31 to 60 min) d) moderate to large amount of activity (1 to 2 h) e) large amount of activity (more than 2 h)	Children who selected either d) or e) (60 min or more) for both weekday and weekends were categorised as being sufficiently active (i.e. meeting guidelines of ≥ 60 min of MVPA) or not	Bespoke deriving of variable using Youth Activity Profile^a^
Sedentary—watching TV Sedentary—playing video games Sedentary—using computers/tablets Sedentary—using mobile phone Sedentary—doing school work	During the last 7 days, on a normal day, how many hours did you spend doing the following activities while sitting or lying down? - watching TV (but not time spent play video games) - playing video games (on a game console e.g. PlayStation or switch, mobile phone, tablet or computer) - using computers/tablets (for social activity, e.g. social media, surfing web or video calling but not playing computer games or school work) - using mobile phone (to talk, text or socialise, e.g. social media, but not playing games - doing school work (in books or on a computer/tablet (e.g. maths, reading, topic work)	a) No activity b)1 h or less c)1–2 h d)3–4 h e) > 4 h	Children were asked to report on a Likert scale (0 = no time, 5 = 4 h or more) how much time they spent on a normal day in the last 7 days being sedentary (sitting, reclining or lying down) whilst watching television, playing video games, using a mobile phone, a computer/tablet. An additional question of ‘*doing school work*’ was also included to capture the amount of time children spent doing sedentary school work during COVID-19 restrictions. Categories were collapsed into < 1 h, 1–3 h, > 3 h, due to the small number of Responses for no activity and 4 h or more	Youth Activity Profile
Sedentary screen time	Variable made up of sedentary TV, video games, using computer/tablets, mobile phone NOT school work	Derived variable	Each category was coded with a time amount (i.e. no time = 0, less than 1 h = 0.5, 1–2 h = 1.5, 3–4 h = 3.5, 4 h = 4)). Summed all of the time amounts to estimate normal time spent using screen. Children with > 2 h as not meeting screen time guidelines = 0, < 2 h meeting ST-guidelines = 1	Bespoke deriving of variable using Youth Activity Profile
Sleep	a) in the last seven days what time have you normally fallen asleep? b) in the last seven days what time have you normally woken up?	Free text	Using the time from each answer a self-reported average sleep time was estimated for each child Time in hours was then coded into either not meeting—less than 9 h, meeting—sleeping between 9–11 h, not meeting – sleeping more than 11 h	Bespoke
Frequency of leaving the home (including garden/yard) a day	*on a normal day in the last 7 days, how many times did you leave your home (away from your house and garden)?*	a) Stayed at home b) left home once a day c) left the home more than once a day	Used categories as asked	Bespoke
Duration of leaving the home (including garden/yard) a day	Children who reported leaving the home were asked - how long did you go for?	a) ≤ 30 min b) 31–60 min c) ≥ 60 min	Used categories as asked	Bespoke
Type of activity—when leaving the home?	Children who reported leaving the home were asked - what did you usually do? (choose all that apply)	a) walk b) run/jog c) scoot/ride bike d) other [free text]	Free text answers were analysed by four trained researchers who assessed and agreed upon the following additional categories: Sports and games and Other (non-active pursuits such as travelling to see family and friends)	Bespoke
Destination of activity—when leaving the home?	Children who reported leaving the home were asked - where did you usually go?	a) Street b) Park c)Non- park greenspace/nature d) Shops e) other [free text]	Free text answers were analysed by four trained researchers who assessed and agreed upon the following additional categories: non-park greenspace/nature (e.g. woods, canals, moors, countryside) and Other neighbourhood areas (all other responses)	Bespoke

^a^Youth Activity Profile – English Child Version—Fairclough, S. J., Christian, D. L., Saint-Maurice, P. F., Hibbing, P. R., Noonan, R. J., Welk, G. J., Dixon, P. M., & Boddy, L. M. (2019). Calibration and Validation of the Youth Activity Profile as a Physical Activity and Sedentary Behaviour Surveillance Tool for English Youth. International journal of environmental research and public health, 16(19), 3711. https://doi.org/10.3390/ijerph16193711

Because of the uniqueness of the COVID-19 lockdown and subsequent reduced opportunities for children to being physically active as they normally would be, children were asked to answer questions on the frequency they normally left the home, the duration they would normally leave for, the type of activities they usually did when leaving the home, and where they usually went (Table 1).

#### Before COVID-19

For the sub-sample of children with available data from before COVID-19, PA levels were measured by children completing the PAQ-C with the support of trained researchers during school time (2017–2019). The PAQ-C is a validated PA seven day recall questionnaire, that measures general levels of MVPA of children aged 8–14 years by assessing participation in different physical activities as well as activity during physical education, lunch break, recess (play time), before school, after school, evenings and weekends [33, 36]. The scoring of the PAQ-C is based upon an average of all questions asked, with a score between 1 (low activity rating) to 5 (high activity rating) [33, 36]. Cut-off values indicating whether children were sufficiently active (relating to cardio-respiratory fitness [37]) were applied (2.7 aggregate score [out of 5] for girls, 2.9 aggregate score [out of 5] for boys).

### Statistical analysis

Descriptive statistics for all variables were generated. Continuous variables were described using mean and standard deviation; categorical variables using counts and proportions. For aim one (whole sample, during COVID-19) univariate statistical tests were performed (Pearson Chi-square tests (χ^2^), with Bonferroni-adjusted *p-*values, independent *t*-tests, one-way analysis of variance and non-parametric alternatives) to examine whether there were differences between the outcome variable (sufficiently active [normally doing 60 min of MVPA a day]- yes or no) and independent variables (meeting sleep guidelines, time spent in sedentary behaviours, frequency and duration of leaving the home, and destination and type of activity outside of the home. Because of the inequalities between sex and ethnic groups, univariate associations were examined between all measures with sex and ethnicity categories. Four multivariable logistic regressions were generated for the outcome (sufficiently active [normally doing 60 min of MVPA a day]- yes or no). The first model included key demographic variables (age, sex, ethnicity, IMD) and whether children still attended school. The second model added the five sedentary behaviours to demographics variables. The third model included the frequency children reported of leaving the home. The fourth and final model included only children who reported leaving the home and included the variable of duration of time away from the home, destination children usually went to, and type of PA children did when away from the home. For aim two change over time from baseline (pre-COVID-19) to follow-up (during COVID-19) for children being sufficiently active (binary 0 for ‘No’ and 1 for ‘Yes’) was investigated using the McNemar test for significance of changes on the subsample of children with data available at the time at different time points (pre-COVID-19, during the first COVID-19 lockdown). Potential demographic factors, associated with any significant change in compliance (sex, age difference [months] between pre-post COVID, ethnicity, IMD) were investigated using logistic regression through simultaneous entry of independent variables. The outcome variable was coded 0 for the ‘absence of negative change’ for being sufficiently active and 1 for the ‘presence of negative change’ being sufficiently active. An alpha value ≤ 0.05 was considered statistically significant. All analysis was conducted using Stata v15.0 (StataCorp., College Station, TX).

## Results

### Descriptive Statistics

Out of a total n = 5,298 eligible children, n = 970 (18.3%) children agreed to take part, completed and returned a survey in spring 2020. A total of n = 949 (17.9%) had completed PA data and were included in the analysis for aim 1. A total of n = 634 (66.8%, based upon 949 children) children had matched PA data prior to COVID-19. The characteristics for both pre and during COVID-19 samples are reported in Table 2.

**Table 2:** Study samples demographics and characteristics

	During COVID-19 sample (n = 949)	Pre-COVID-19 sample (n = 643)
**Age**, m (SD)^a^	10.5 (1.1)	9.1 (1.1)
**Sex**, n (%)		
Male	486 (51.2)	321(50.6)
Female	463 (48.8)	313 (49.4)
**Ethnicity**, n (%)		
White British	385 (40.6)	254 (40.1)
Pakistani Heritage	418 (44.1)	275 (43.4)
Other Ethnicity	146 (15.4)	105 (16.6)
**Index of Multiple Deprivation**, n (%)		
Most deprived nationally	355 (37.4)	237 (37.4)
2nd most deprived nationally	140 (14.8)	97 (15.3)
3rd most deprived nationally	166 (17.5)	114 (18.0)
4th < most deprived nationally	288 (30.5)	186 (29.3)
**Attending Schoo**l, n (%)		
Yes	95 (10.0)	563 (89.4)
No	854 (90.0)	67 (1.6)

^a^The average increase age between pre and during COVID-19 first lockdown was 1.2 years (0.72) /15.2 months (8.6)

### During COVID-19 lockdown: Levels of self-reported physical activity and activity away from the home

Twenty-seven per cent of children reported being sufficiently active (> 60 min MVPA daily) during the first COVID-19 lockdown (Table 3). Children reported spending an average of 10.6 h (SD = 1.5) a day sleeping, with 69% meeting sleep guidelines of 9 to 11 h/day. Almost one third of children reported spending ≥ 3 h a day doing sedentary schoolwork (32.9%) and ≥ 3 h a day playing sedentary video games (29.6%), and a majority of children did not meet screen time guidelines (89.9%). The majority of children reported that they had usually left the home environment during the previous seven days, with 53.9% leaving once a day, and 16.7% more than once a day. However, 30% reported that they had normally stayed at home. Of the children who reported leaving the home at least once a day, the majority of children reported leaving between 31–60 min (54%). The most frequently reported type of activities outside of the home was walking (77%) and riding a bike/scooter (41.9%), and the most frequent reported places for children to go was the street (33.7%) and park (34.5%).

**Table 3:** Levels, sex and ethnic differences of children’s self-reported physical activity, usual sleep duration, sedentary behaviours, whether attending school, frequency and duration of leaving the home environment during a COVID-19 UK restrictions (April-June 2020)

	**All (n = 946)**	**Males (n = 486)**	**Females (n = 463)**	***p***	**White British (n = 385)**	**Pakistani Heritage (n = 418)**	**Other (n = 146)**	***p***
**Physical activity self-reported during COVID-19 first lockdown (MVPA—≥ 60 min daily)**, n (%)								
Sufficiently active	259 (27.4)	145 (29.8)	114 (24.8)	.08	131 (34.1)	95 (22.8)	33 (22.8)	**.00**
Not sufficiently active	687 (72.6)	341 (70.2)	246 (75.2)		253 (65.9)	322 (77.2)	112 (77.2)	
**Attending Schoo**l, n (%)								
Yes	95 (10.0)	56 (11.5)	39 (8.4)	.11	54 (14.0)	25 (6.0)	16 (10.7)	**.00**
No	854 (90.0)	430 (88.5)	424 (91.6)		331 (86.0)	393 (94.02)	130 (89.0)	
**Meeting Sleep guidelines**, n (%)								
Not meeting—less than 9 h	63 (6.8)	39 (8.3)	24 (5.3)	**.00**	29 (7.7)	22 (5.5)	12 (8.5)	**.00**
Meeting guidelines—9–11 h	637 (68.9)	336 (71.3)	301 (66.5)		303 (80.2)	239 (59.1)	95 (66.9)	
Not meeting—Sleep more than 11 h	224 (24.2)	96 (20.4)	128 (28.3)		46 (12.1)	143 (35.4)	35 (24.6)	
**Sedentary- Watching Television** (not time playing video games), n (%)								
< 1 h	393 (42.3)	211 (44.4)	182 (40.0)	.26	160 (42.7)	173 (41.9)	60 (42.3)	.46
1–3 h	380 (40.9)	182 (38.3)	198 (43.5)		160 (42.7)	160 (38.7)	60 (42.3)	
3 h <	157 (16.9)	82 (17.3)	75 (16.5)		55 (14.6)	80 (19.4)	22 (15.4)	
**Sedentary—Video games on a games console**, n (%)								
< 1 h	365 (38.9)	124 (25.8)	241 (52.6)	**.00**	117 (30.7)	176 (42.7)	72 (49.7)	**.00**
1–3 h	295 (31.5)	157 (32.7)	138 (30.1)		120 (31.5)	137 (33.3)	38 (26.2)	
3 h <	278 (29.6)	199 (41.5)	79 (17.3)		144 (37.8)	99 (24.0)	35 (24.1)	
**Sedentary—Computers/tablets use for social activity**, n (%)								
< 1 h	653 (70.6)	338 (71.9)	315 (69.23)	.56	259 (69.4)	283 (69.0)	111 (78.2)	.309
1–3 h	176 (19.03)	83 (17.7)	93 (20.4)		74 (19.8)	81 (19.8)	21 (14.8)	
3 h <	96 (10.38)	49 (10.4)	47 (10.3)		40 (10.7)	46 (11.2)	10 (7.0)	
**Sedentary—Mobile phone use** (not playing games), n (%)								
< 1 h	707 (76.4)	386 (81.4)	321 (71.0)	**.00**	224 (64.9)	342 (83.4)	121 (86.4)	**.00**
1–3 h	123 (13.3)	55 (11.6)	68 (15.0)		78 (20.7)	32 (7.8)	13 (9.3)	
3 h <	96 (10.4)	33 (7.0)	63 (13.9)		54 (14.4)	36 (8.8)	6 (4.3)	
**Sedentary—School Work** (books, computers), n (%)								
< 1 h	271 (28.8)	143 (29.7)	128 (27.9)	.01*	100 (26.3)	134 (32.4)	37 (25.3)	**.00**
1–3 h	360 (38.3)	201 (41.8)	159 (34.6)		128 (33.7)	170 (41.1)	62 (42.5)	
3 h <	309 (32.87)	137 (28.5)	172 (37.5)		152 (40.0)	110 (26.6)	47 (32.2)	
**Screen time—Meeting Guidelines ≤ 2 Hours**								
Not Meeting	842 (89.9)	438 (91.4)	404 (88.2)	.10	353 (93.6)	364 (87.3)	125 (87.4)	**.00**
Meeting	95 (10.1)	41 (8.6)	54 (11.8)		24 (6.4)	53 (12.7)	18 (12.6)	
**Frequency of leaving home (including garden/yard) a day**, n (%)								
Stayed at home	279 (29.7)	145 (30.0)	134 (29.4)	.10	68 (17.9)	163 (39.5)	48 (32.9)	**.00**
Once a day	507 (53.9)	260 (53.7)	247 (54.2)		244 (64.0)	193 (46.7)	70 (48.0)	
More than once a day	154 (16.4)	79 (16.3)	75 (16.5)		69 (18.1)	57 (13.8)	28 (19.2)	
**Duration of time away from home (including garden/yard) a day**, n(%)								
< 30 min	100 (15.0)	44 (12.9)	56 (17.2)	.17	30 (9.6)	56 (22.1)	14 (14.3)	**.00**
31–60 min	359 (54.0)	182 (53.5)	177 (54.5)		163 (51.9)	141 (55.7)	55 (56.1)	
60 min <	206 (31.0)	114 (33.5)	92 (28.3)		121 (38.5)	56 (22.1)	29 (29.6)	
**Destination of activity—when leaving the home** n(%)								
Street	221 (33.7)	106 (31.2)	115 (36.4)	.37	101 (32.6)	91 (36.7)	29 (29.9)	**.00**
Shops	64 (9.8)	31 (9.1)	33 (10.4)		22 (7.1)	31 (12.5)	11 (11.3)	
Park	226 (34.5)	118 (34.8)	108 (34.2)		83 (26.8)	103 (41.5)	40 (41.2)	
Non-park greenspace (e.g. woods, local fields)	80 (12.2)	36 (10.6)	28 (8.9)		67 (21.6)	6 (2.4)	7 (7.2)	
Other neighbourhood areas	64 (9.8)	48 (14.2)	32 (10.1)		37 (11.9)	17 (6.9)	10 (10.3)	
**Type of activity—when leaving the home- Walk** n(%)								
No – did not walk	154 (23.1)	93 (27.1)	61 (18.9)	**.01**	54 (17.1)	75 (29.5)	25 (25.3)	**.00**
Yes – did walk	514 (77.0)	250 (72.9)	264 (81.2)		261 (82.9)	179 (70.5)	74 (74.8)	
**Type of activity—when leaving the home—Run/Jog** n(%)								
No – did not Run/Jog	535 (80.1)	263 (76.7)	272 (83.7)	.02*	259 (82.2)	207 (81.5)	69 (69.7)	.02*
Yes – did Run/Jog	133 (19.9)	80 (23.3)	53 (16.3)		56 (17.8)	47 (18.5)	30 (30.3)	
**Type of activity—when leaving the home – Ride bike/scoot** n(%)								
No – did not Ride bike/scoot	388 (58.1)	187 (54.5)	201 (61.9)	.06	171(54.3)	164 (64.6)	53 (53.5)	.03*
Yes – did Ride bike/scoot	280 (41.9)	156 (45.5)	124 (38.1)		144 (45.7)	90 (35.4)	46 (46.5)	
**Type of activity—when leaving the home –Play, Sports or Games** n(%)								
No – did not play sports or games	612 (91.6)	308 (89.8)	304 (93.5)	.08	296 (94.0)	230 (90.6)	86 (86.9)	.06
Yes – did play sports or games	56 (8.4)	35 (10.2)	21 (6.5)		19 (33.9)	24 (9.5)	13 (13.1)	
**Type of activity—when leaving the home – Other (e.g. travelling in car)** n(%)								
No – did not do ‘other’	636 (95.2)	330 (96.2)	306 (94.2)	.21	305 (96.8)	238 (93.7)	93 (93.9)	.18
Yes – did do ‘other’	32 (4.8)	13 (3.8)	19 (5.9)		10 (3.2)	16 (6.3)	6 (6.1)	

^*^ Non-significant due to Bonferroni correction

### During COVID-19 lockdown: Sex and ethnicity behaviour differences during the COVID-19 lockdown

Univariate sex and ethnicity differences are reported in Table 3. Differences between boys and girls were found for sleep duration (≥ 11 h: Girls = 28.3% > Boys = 20.4%); time spent normally playing console video games (≥ 3 h: Boys = 41.5% > Girls = 17.3%); using mobile phones (≥ 3 h: Girls = 13.9% > Boys = 7.0); and usually walking (type of activity) when outside of the home (Girls = 81.2% > Boys = 72.9%). Significant differences between ethnic groups were found for being sufficiently active (WB = 34.1% vs PH = 22.8% vs O = 22.8%), still attending school (WB = 14% vs. P = 6% and O = 10.7%); sleep duration (9–11 h: WB = 80.2% vs PH = 59.1% vs O = 66.9); time spent normally—playing console video games (≥ 3 h:WB = 37.8% vs PH = 24.% vs O = 24.1%), using mobile phones (≥ 3 h: WB = 14.4% vs PH = 8.8% vs O = 4.3%), meeting ST-guidelines (< 2 h WB = 6.4% vs PH = 12.7% vs 12.6%); frequency of leaving the home (stayed at home: PH = 39.5% vs O = 32.9% vs WB = 17.9%); duration of time leaving the home (≥ 60 min: WB = 38.5% vs PH = 22.1% vs O = 29.6%); places children usually went outside of the home (Park: PH and O = 41.5% vs WB = 26.8%, Greenspace/nature: WB = 21.6% vs PH = 2.4% vs O = 7.2%); and usually walking when outside of the home (WB = 82.9% vs PH = 70.5% vs O = 74.8%).

### During COVID-19 lockdown: Factors associated with children being sufficiently active during COVID-19 lockdown

Univariate factors between children’s self-reported PA and predictor variables (Table 4) were age, ethnicity, duration of playing video games on a console, normal daily frequency of leaving the home, normal daily duration of leaving the home, the place children usually went to outside of the home, and if they took part in running/jogging, riding a bike/scooter, and playing sports and games.

**Table 4:** Univariate analysis of difference between children sufficiently physically active (> 60 min usually a day) with demographics, and independent variables during COVID-19 UK restrictions

	Sufficiently physically active		*p*
	**Yes n = 259 (27.4%)**	**No n = 687 (72.6%)**	
**Age**, m (SD)	10.3 (1.1)	10.6 (1.1)	**.01**
**Gende**r, n (%)			
Male	145 (29.8)	341 (70.2)	.08
Female	114 (24.8)	246 (75.2)	
**Ethnicity**, n (%)			
White British	131 (34.1)	253 (65.9)	**.00**
Pakistani Heritage	95 (22.8)	322 (77.2)	
Other ethnicities	33 (22.8)	112 (77.2)	
**Index of Multiple Deprivation**, n (%)			
Most deprived nationally	81 (22.9)	273 (77.1)	.02*
2nd most deprived nationally	43 (30.7)	97 (69.3)	
3rd most deprived nationally	40 (24.2)	125 (75.8)	
4th < most deprived nationally	95 (33.1)	192 (66.9)	
**Attending School**, n (%)			
Yes	34 (35.8)	61 (64.21)	.05
No	225 (26.4)	626 (73.7)	
**Meeting Sleep guidelines—self reported**, n (%)			
Not meeting—less than 9 h	16 (25.4)	47 (74.6)	.01**
Yes- meeting guidelines—9–11 h	193 (30.4)	443 (69.7)	
Sleep more than 11 h	45 (20.1)	170 (80.9)	
**Watching Television** (not time playing video games), n (%)			
< 1 h	112 (28.6)	219 (71.4)	.04***
1–3 h	111 (29.3)	268 (70.7)	
3 h <	30 (19.1)	127 (80.9)	
**Video games on a games console**, n (%)			
< 1 h	109 (30.0)	155 (70.0)	**.00**
1–3 h	95 (32.4)	198 (67.6)	
3 h <	53 (19.1)	225 (80.9)	
**Computers/tablets use for social activity**, n (%)			
< 1 h	189 (28.9)	466 (71.2)	.12
1–3 h	49 (27.8)	127 (72.2)	
3 h <	18 (28.7)	79 (81.4)	
**Mobile phone use** (not playing games), n (%)			
< 1 h	199 (28.2)	506 (71.8)	.14
1–3 h	36 (29.3)	87 (13.0)	
3 h <	18 (19.0)	77 (11.5)	
**School Work** (books, computers), n (%)			
< 1 h	69 (25.3)	204 (74.7)	.10
1–3 h	90 (25.0)	270 (75.0)	
3 h <	100 (32.3)	210 (67.7)	
**Screen time—Meeting Guidelines ≤ 2 Hours**			
Not Meeting	72 (10.6)	607 (89.4)	.38
Meeting	232 (8.7)	232 (91.3)	
**Frequency of leaving home (including garden/yard) a day**, n (%)			
Stayed at home	46 (16.6)	232 (83.5)	**.00**
Once a day	149 (29.4)	358 (70.6)	
More than once a day	62 (40.3)	92 (59.7)	
**Duration of time away from home (including garden/yard) a day**, n(%)			
< 30 min	9 (9.0)	91 (91.0)	**.00**
31–60 min	91 (25.4)	268 (74.7)	
60 min <	111 (53.9)	95 (46.1)	
**Destination of activity—when leaving the home** n(%)			
Street	66 (29.9)	155 (70.1)	**.00**
Shops	6 (9.4)	58 (90.6)	
Park	77 (34.2)	148 (65.8)	
Greenspace/nature (e.g. woods, local fields)	34 (42.5)	46 (57.5)	
Other neighbourhood areas	27 (42.2)	37 (57.8)	
**Type of activity—when leaving the home—Walk** n(%)			
No – did not walk	47 (30.5)	107 (69.5)	.70
Yes – did walk	165 (32.2)	348 (67.8)	
**Type of activity—when leaving the home – Run/Jog** n(%)			
No – did not Run/Jog	155 (29.0)	379 (70.1)	**.00**
Yes – did Run/Jog	57 (42.9)	76 (57.1)	
**Type of activity—when leaving the home – Ride bike/scoot** n(%)			
No – did not Ride bike/scoot	99 (25.8)	288 (74.4)	**.00**
Yes – did Ride bike/scoot	113 (40.4)	167 (59.6)	
**Type of activity—when leaving the home –Play, Sports or Games (e.g. playing** n(%)			
No – did not play sports or games	186 (30.4)	425 (69.6)	**.01**
Yes – did play sports or games	26 (46.4)	30 (53.6)	
**Type of activity—when leaving the home – Other things (e.g. travel in car)** n(%)			
No	205 (32.3)	430 (67.7)	**.01**
Yes	7 (21.9)	25 (78.1)	

* corrected *p*-value = .006, non-significant

** corrected *p*-value = .008, non-significant

*** corrected *p*-value = .008, non-significant

For the multivariable analysis, summaries of logistic regression models (1, 2, 3, 4) are reported in Table 5 (a full results table is found in Additional file 1: Appendix 1 – supplementary material). In model 1, variables that decreased the odds of being sufficiently active were age (years) (Odds ratio, OR = 0.82, 95%CI 0.72–0.94), and ethnicity (reference: WB); PH children (OR 0.64, 95%CI 0.44–0.92), Other (OR = 0.57, 95%CI 0.35–0.90). In model 2 (which included sedentary behaviours), being a girl (OR = 0.63, 95%CI 0.45–0.88) and playing on video games ≥ 3 h a day (OR = 0.43, 95%CI 0.28–0.67) significantly decreased the odds of being sufficiently active, in addition to age and ethnicity. In model 3 (which included daily frequency of leaving the home), age, being a girl, being from another ethnic group and playing video games (≥ 3 h a day) still decreased the odds of being sufficiently active; however, being of PH no longer did. Leaving the home at least once a day significantly increased the odds (OR = 1.57 95%CI(1.04–2.36)), with the odds increasing further for children who reported leaving the home more than once a day (OR = 2.73, 95%CI 1.66–4.48). In model 4, (which included duration, place and type of activity), age and playing videos for ≥ 3 h/day significantly decreased the odds, but leaving the home for 31–60 min significantly increased the odds (OR = 2.21, 95%CI 1.01–4.8), and the odds increased further for children reporting leaving the home for ≥ 60 min (OR = 7.9, 95%CI 3.5–18.0). Children reporting that the place they usually went too was the shop which reduced the odds of children being sufficiently active (OR = 0.36, 95%CI 0.13–0.98). Odds were increased for children reporting that usually took part in running/jogging (OR = 2.13, 95%CI 1.30–3.47), riding a bike/scooter (OR = 1.52, 95%CI 1.01–2.31), and playing sports and games (OR = 2.13, 95%CI 3.4–2.70).

**Table 5:** Multivariable logistic regression analysis of factors (demographic, self-reported sleep duration and sedentary behaviours, school attendance, frequency, duration, type of activity and place of destination of children when leaving the home environment), with children self-reporting being sufficiently physically active (> 60 min usually a day) during COVID-19 UK restrictions (April-June 2020)

	**Model 1 (n = 946)**			**Model 2 (n = 875)**			**Model 3 (n = 868)**			**Model 4 (n = 602)**		
	OR	(95% CI)	*p*	OR	95% CI	*p*	OR	95% CI	*p*	OR	95% CI	*p*
**Age** (years)	0.82	(0.72–0.94)	**0.00**	0.83	(0.72–0.97)	**0.02**	0.83	(0.12–0.97)	**0.02**	0.81	(0.67–0.99)	**0.04**
**Sex**- Male (Reference)												
Female	0.81	0.60–1.08	0.15	0.63	(0.45–0.88)	**0.01**	0.61	(0.44–0.86)	**0.01**	0.76	(0.50–1.17)	0.22
**Ethnicity—**White British (Reference)												
Pakistani Heritage	0.64	(0.44–0.92)	**0.02**	0.62	(0.41–0.95)	**0.03**	0.72	(0.47–1.12)	0.14	0.82	(0.46–1.48)	0.51
Other	0.57	(0.35–0.90)	**0.02**	0.50	(0.30–0.83)	**0.01**	0.53	(0.31–0.90)	**0.02**	0.48	(0.24–0.94	**0.03**
**Index of Multiple Deprivation** Most Deprived (Reference)												
2nd most deprived	1.46	(0.94–2.28)	0.09	1.30	(0.81–2.08)	0.28	1.22	(0.76–1.98)	0.41	1.41	(0.78–2.54)	0.26
3rd most deprived	0.97	(0.62–1.52)	0.91	0.78	(0.48–1.26)	0.31	0.82	(0.51–1.33)	0.43	0.84	(0.45–1.57)	0.58
4th < most deprived	1.33	(0.89–2.00)	0.17	1.13	(0.72–1.75)	0.60	1.12	(0.71–1.75)	0.63	1.40	(0.81–2.41)	0.23
**Attending School**—Yes (Reference)												
No	1.41	(0.89–2.23)	0.14	1.32	(0.79–2.18)	0.29	1.16	(0.69–1.96)	0.57	1.33	(0.72–2.47)	0.36
**Meeting Sleep guidelines** Not meeting (Reference)												
Yes- meeting (9–11 h)				1.32	(0.69–2.54)	0.40	1.31	(0.67–2.55)	0.43	1.59	(0.69–3.73)	0.29
11 h <				0.87	(0.42–1.81)	0.72	0.88	(0.42–1.13)	0.74	1.43	(0.55–3.70)	0.47
**Watching Television** < 1 h (Reference)												
1–3 h				1.04	(0.75–1.46)	0.80	1.08	(0.77–1.52)	0.65	1.17	(0.76–1.79)	0.48
3 h <				0.74	(0.46–1.19)	0.21	0.69	(0.43–1.23)	0.14	0.74	(0.41–1.33)	0.31
**Video games on a games console** < 1 h (Reference)												
1–3 h				0.91	(0.63–1.32)	0.62	0.92	(0.63–1.33)	0.67	0.94	(0.58–1.51)	0.95
3 h <				0.43	(0.28–0.67)	**0.00**	0.45	(0.29–0.70)	**0.00**	0.52	(0.30–0.90)	**0.02**
**Computers/tablets use for social activity** < 1 h (Reference)												
1–3 h				1.11	(0.74–1.68)	0.61	1.08	(0.71–1.63)	0.72	1.34	(0.81–2.33)	0.25
3 h <				0.43	(0.44–1.45)	0.46	0.86	(0.47–0.71)	0.64	1.67	(0.72–3.86)	0.45
**Mobile phone use** < 1 h (Reference)												
1–3 h				1.03	(0.64–1.66)	0.90	1.11	(0.69–1.79)	0.68	1.04	(0.58–1.89)	0.88
3 h <				0.73	(0.39–1.37)	0.33	0.81	(0.43–1.52)	0.51	0.73	(0.31–1.68)	0.45
**School Work** < 1 h (Reference)												
1–3 h				0.89	(0.60–1.32)	0.56	0.89	(0.59–1.32)	0.56	0.85	(0.51–1.41)	0.52
3 h <				1.20	(0.80–1.80)	0.37	1.20	(0.79–1.81))	0.39	1.03	(0.61–1.73)	0.91
**Frequency of leaving home** Stayed at Home (Reference)												
Once a day (Reference—Model 4)							1.57	(1.04–2.36)	**0.03**			
More than once a day							2.73	(1.66–4.48)	**0.00**	1.08	(0.67–1.73)	0.75
**Duration away from home** < 30 min (Reference)												
31–60 min										2.21	(1.01–4.8)	**0.04**
60 min <										7.9	(3.5–18.0)	**0.00**
**Destination of activity—when leaving the home?** Street (reference)												
Shops										0.36	(0.13–0.98)	**0.04**
Park										1.09	(0.68–1.77)	0.72
Greenspace/nature										0.97	(0.49–1.88)	0.92
Other neighbourhood areas										0.90	(44.3–1.85)	0.78
**Type of activity—when leaving the home, Walk**—No (reference) Yes										1.15	(0.70–1.88)	0.59
**Type of activity—when leaving the home, Run/Jog** - No (reference) Yes										2.13	(1.30–3.47)	**0.00**
**Type of activity—when leaving the home, Ride bike/scoot** - No (reference) Yes										1.52	(1.01–2.31)	**0.04**
**Type of activity—when leaving the home, Play, Sports or Games**—No (reference) Yes										2.13	(1.1–4.31)	**0.03**
**Type of activity—when leaving the home, Other**—No (reference Yes)										0.96	(0.34–2.70)	0.93
Constant	3.67	(0.86–15.64)	0.078	4.39	(0.78–24.83)	0.09	2.64	(0.44–15.85)	0.29	0.6748692	(0.06–7.25)	0.75
Log likelihood	-539.2033			-483.34			-471.39388			-310.63993		
Pseudo r-square	0.029			0.062			0.077			0.1758		
Likelihood-Ratio chi-square (df)	32.17 (8), *p* = 0.000			63.34 (20), *p* = .0001			78.85(22), *p* = .0000			106.86(32), *p* = 0.000		

### Changes in children being sufficiently physically active before and during the COVID-19 lockdown

The sub-sample’s pre-COVID-19 PA (sub-sample, n = 643) was PAQ-C score 3.2 (SD = 0.77), with 69.4% (n = 440) found to be sufficiently active. During COVID-19 the proportion of children being sufficiently active reduced to 28.7% (n = 183). The magnitude of change was statistically significant (see Table 6), with 47.5% of children changing from being sufficiently active before COVID-19 to not being sufficiently active during COVID-19. A small number of children who were not sufficiently active pre-COVID did report being sufficiently active during COVID-19 (7.0%, n = 44), leading to a 40.5% reduction. A logistic regression model (Table 7) predicted that the age difference between the two measurement periods, ethnicity and sex did not significantly increase or decrease the odds of children negatively changing from being sufficiently active from before COVID-19 to during COVID-19.

**Table 6:** McNemar test for significance of changes in reported physical activity before COVID-19 and during COVID-19

Baseline	Follow-Up		McNemar test statistic		
	Sufficiently active	Not sufficiently active	*Χ*^2^	df	*P*
Sufficiently active	139 (21.8%)	301 (47.5%)	191.5	1	0.00*
Not sufficiently active	44 (7%)	150 (23.7%)

**Table 7:** Factors for change in children being sufficiently physically active measured by self-report before and during COVID-19

	negative change vs. no change/positive change **n = 634**		
	OR	(95% CI)	*p*
**Age difference** (months)	1.0	(0.99–1.4)	0.09
**Sex**- Male (Reference)			
Female	0.9	(0.65–1.2)	0.50
**Ethnicity—**White British (Reference)			
Pakistani Heritage	1.2	(0.80–1.80)	0.36
Other ethnicities	1.2	(0.77–2.02)	0.37
**Index of Multiple Deprivation**—most deprived			
2nd most deprived nationally	0.7	(0.43–1.11)	0.14
3rd most deprived nationally	1.1	(0.66–1.67)	0.69
4th < most deprived nationally	0.8	(0.51–1.24)	0.44
Constant	1.1	(0.77–2.02)	0.83
Log likelihood	-425.77		
Pseudo r-square	0.027		
Likelihood-Ratio chi-square (df)	23.43 (7), *p* = 0.0014		

## Discussion

The purpose of this study was to investigate the levels, factors associated and change of children’s self-reported PA during the first COVID-19 in England. Results show levels of children reporting being sufficiently active has drastically reduced from before COVID-19. Factors associated with meeting guidelines during the first COVID-19 lockdown were child’s age, ethnicity (Pakistani Heritage and Other ethnic minorities [-]), sex (girls), self-reported video game usage (> 3 h a day[-]), and the frequency (> 1 a day[ +]), duration (> 31 min[ +]), type of activity (run/jog, ride bike/scooter, play, sports or games [ +]) and place visited when leaving the home environment (shops [-]).

Only a quarter of children reported being sufficiently active enough to benefit their health during the first COVID-19 lockdown, and this reduced greatly from before COVID-19, independently of increased age. These findings are similar to other studies [19, 21, 38] and are unsurprising when considering the sharp change in the systems in which children’s PA would usually occur (i.e. school, sport clubs, parks, playgrounds, active travel). Daily PA outside of the home environment was allowed and has been consistently allowed by the UK-government during the first lockdown and throughout the pandemic, but not actively promoted [38, 39]. This is unsurprising due to the priority being to reduce mixing of individual households. As the BiB COVID-19 study [26] progresses further studies will be able to report on changes in PA during the pandemic and during differing restriction circumstances. It is likely, given the ongoing restrictions, that PA levels will remain lower than pre-pandemic. The short- and long-term health implications for reduced PA for a sustained period of time during childhood is unknown and this is something which the cohort study aims to investigate. Early life is particularly important for habit formation and has been shown that PA tracks from across the life course of young people [40–42] so there is a possibility of long term health implications associated with reduced PA across the lifespan, triggered by reduced PA during the COVID-19 pandemic. This is something that requires careful monitoring and preventative interventions to reduce the likelihood of ongoing low PA levels.

Previous non-pandemic research has shown an association between children’s PA levels and time spent outdoors, [43, 44] with the current study’s findings highlight how important time away from the home environment was for being active. Worryingly, 29.7% of children reported that they didn’t leave the home on a usual day during lockdown and this was strongly associated with not being sufficiently active (OR = 1.6 once a day, OR = 2.7 ≥ once a day). For those who did leave the home, just under half of children (46%) did so for longer than 60 min and leaving the home environment for this amount of time was found to be important for children being sufficiently active (i.e. MVPA-60 min guidelines, OR = 7.9). The government guidance during the first and all subsequent lockdowns (November 2020, January—currently 2021) has been to minimise the time spent outside of the home, and there has been a common misconception that exercising away from the home should be for no longer than one hour [45]. This study illustrates the importance of extending the amount of time away from the home for children to be physically active, and if this can be done safely, should be promoted.

The places children most frequently reported going to were, the streets, and parks, and the most frequent activities reported were walking and riding a bike/scooter. The results showed that children who reported going to the shops had reduced odds of being sufficiently active, therefore illustrating that getting out of the home environment to places which are conducive to being active (e.g. streets, parks, greenspaces) should be encouraged, whilst adhering to current COVID-19 guidelines and taking always necessary precautions (e.g. staying 2 m apart). Furthermore, as would be expected, children who reported engaging in more vigorous PA such as riding a bike/scooter and playing sports and games were more likely to be sufficiently active than those who reported just walking; suggesting that campaigns should focus on the promotion of these more vigorous types of activities, but also acknowledge that any PA is worthwhile and should be promoted.

There were large differences in whether children reported leaving the home and for how long between ethnic groups. PH and O children left the home significantly less often than their WB peers and for shorter periods. When frequency of leaving the home was controlled for, ethnic PA differences no longer existed between WB and PH children, therefore highlighting an inequality in a key factor for why children were sufficiently active during COVID-19 lockdown (i.e. more PH children stayed at home than WB, therefore were less active). Because of the importance of leaving the home environment to be sufficiently active during COVID-19 lockdown, it is important for policy, strategy and practice to consider why some children were leaving the home environment and why others were not, particularly between different ethnic groups. The current study did not directly ask children to report why they had not left their home so this could not be examined. It may have been that those who did not leave the home environment were living in areas less conducive for PA. The following environmental determinants of children and adolescents PA have previously been identified: walkability, availability/access/proximity to recreational facilities, environment aesthetics, negative street characteristics [46]. All such determinants were not explored in the current study and should be considered in future research to possibly explore the ethnic differences found.

A further influence upon whether children were leaving the home in the current study may have been worries and stress experienced by families during lockdown. Mothers of the children from the sample of this study, who mostly live in areas of high deprivation reported numerous difficulties during the spring 2020 lockdown with many insecurities (financial, employment, housing, clinical symptoms of anxiety and depression) and high levels of anxiety about becoming ill or dying from COVID-19 [47] furthermore, COVID-19 has disproportionately impacted ethnic minority groups such as Pakistani, South Asian, and Black ethnicities more than White British, with greater ill health and death reported [47]. Anxiety and fear of ill health and death could be greater within PH and O groups leading to them not wanting to leave the home environment. Negative mainstream media reporting on ethnic minorities violating lockdown protocols and government guidelines, and fear of getting labelled when outside of home could have been another reason for ethnic minority children not leaving home during lockdown. More research using qualitative and anthropological methodologies are required to begin to understand this complex phenomenon, because there is a risk of the exacerbation of PA inequalities between ethnic groups, which were well established pre-COVID-19 [4, 16, 23, 48].

The guidance to stay at home during lockdown periods and anxieties surrounding leaving the home, and the association between leaving the house and physical activity, has created a demand for home-based PA interventions for children, with numerous options being made available [49, 50]. Previous research on the determinants of children’s home PA are unclear with inconsistent findings [51]. These programmes which have been developed rapidly may not be evidence based or grounded in behaviour change theory. Moreover, there is a dearth of literature regarding the feasibility, acceptability, efficacy and effectiveness of such home-based PA programmes/interventions [52]. Home-based PA will likely remain in demand for the foreseeable future as part of the gradual reopening of society, and the changing of the home environment from one promoting mainly sedentary time activities to more physically active activities [51] is a topic of priority to further understand how best is it for children to be active within their home environments.

A concern of the COVID-19 lockdown(s) has been a possible increase of sedentary (particularly screen behaviours) and disturbances of sleep [18, 53–55]. Findings from this study showed that the majority of children reported meeting sleep guidelines (9–11 h a day), engaged in more than one hour a day of TV viewing, playing video games on a console, and doing school work; but also only a small proportion of children (10.1%) reported in meeting ST-guidelines recommendations. Data from Canadian young people also found similar low levels of ST compliance during the first COVID-19 lockdown (11.3%) [19]. But the current study has most likely underestimated the amount of ST, due to the use of a screen for school work (school work item queried any school work, whether using a screen or not) not being factored in the estimate. The high non-compliance of ST recommendations is higher compared to a UK sample of young people pre-COVID (23.1%) [56], which is unsurprising for children restricted to the home environment for much of their time. Of all of the sedentary ST behaviours, playing a video game on a console for ≥ 3 h decreased the odds (OR = 0.43–0.52) of children being sufficiently active. However, it must be noted that significantly more boys reported playing video games for more than 3 h a day than girls, and this may explain why there was not a found difference between the sexes, which is usually commonly found in physical activity data. These findings suggests that alongside promotion of leaving the home to support PA during and following the pandemic, reducing the use of sedentary ST, in particular video game usage (especially for boys) also needs addressing by public health campaigns. The ethnic and sex differences of sleep and sedentary behaviours (video games, mobile usage, school work) found in this study should be further explored with different outcomes such as educational, emotional and mental health which all have been associated previously with sleep and sedentary behaviours [57].

The limitations of this study include use of two different child self-reported PA questionnaires (for pre and during COVID) with one questionnaire (amended- YAP) not being formally validated. The BiB study had previously used the validated PAQ-C questionnaire due to availability of children’s questionnaires for previous cohort data collection [25], before the YAP had been published. In the current study, the PAQ-C was decided by authors not to be suitable for use during lockdown with the majority of children not attending school and being restricted to their homes. Irrespective of the questionnaires used, an obvious limitation of this study is the use of children’s self-reported behaviour and recall. A further limitation is that causality of the variables associated with PA-guidelines cannot be implied, and neither can the direction of association, this is due to the cross-sectional nature of data presented. However, the circumstances of COVID-19 and the ability to rapidly survey and receive data from 979 children and continue to follow and collect further data in the future, is a strength of this city-wide cohort study. The ongoing study is providing insights into the lives of children and families during an ongoing pandemic and provide scientific insight for policy makers to make evidence informed decisions and guidance [26, 58].

## Conclusion

The findings of this study are important for practitioners, policy and decision makers to consider in order to begin to understand the impact and consequences that the drastic but required COVID-19 measures (i.e. lockdown) has had upon children’s PA which is a key and vital behaviour for health and development. Key associations have been identified between self-reported PA and the frequency and length of time children went outside of the home. COVID-19 guidelines should factor that many children will not be sufficiently active just in the home environment. Leaving the home for physical activity/exercise for a minimum of 60 min, and preferably longer each day safely (staying in household bubbles, social distancing, wearing face coverings where necessary) should be actively prioritised and promoted through campaigns and initiatives. Findings should be considered now during the ongoing COVID-19 crisis to support children's PA and short-term health and wellbeing; and, once COVID-19 is under control. Policies and interventions to facilitate ‘recovery’ after COVID-19 will be required to prevent potential long-term health problems associated with low levels of PA during the pandemic.

## Supplementary Information

**Additional file 1.**

**Additional file 2.**

## Data Availability

The datasets used and/or analysed during the specific current study are available from the corresponding author on reasonable request. Scientists are encouraged and able to use BiB data, which are available through a system of managed open access. The steps below describe how to apply for access to BiB data. ● Before you contact BiB, please make sure you have read our Guidance for Collaborators (https://borninbradford.nhs.uk/research/guidance-for-collaborators) Our BiB executive review proposals on a monthly basis and we will endeavor to respond to your request as soon as possible. You can find out about the different datasets which are available (https://borninbradford.nhs.uk/research/documents-data/). If you are unsure if we have the data that you need please contact a member of the BiB team (borninbradford@bthft.nhs.uk). ● Once you have formulated your request please complete the ‘Expression of Interest’ form available (https://borninbradford.nhs.uk/wp-content/uploads/Expression-of-interest-proforma-v2_DM-12.01.18.doc) and send to borninbradford@bthft.nhs.uk ● If your request is approved we will ask you to sign a collaboration agreement https://borninbradford.nhs.uk/wp-content/uploads/BiB_CollaborationAgreement-12.01.18.docx and if your request involves biological samples we will ask you to complete a material transfer agreement https://borninbradford.nhs.uk/wp-content/uploads/BiB_CollaborationAgreement-12.01.18.docx.

## Abbreviations

COVID-19/ COVID: Coronavirus disease 2019
SARS-CoV-2: Severe acute respiratory syndrome coronavirus 2
BiB: Born in Bradford
IMD: Index of multiple deprivation
PA: Physical activity
MVPA: Moderate-to-vigorous physical activity
OR: Odds Ratio
ST: Screen time
WB: White British
PH: Pakistani heritage
O: Other
YAP: Youth Activity Profile
PAQ-C: Physical Activity Questionaire – Children
